# Histone H3K27 acetylation is dispensable for enhancer activity in mouse embryonic stem cells

**DOI:** 10.1186/s13059-020-01957-w

**Published:** 2020-02-21

**Authors:** Tiantian Zhang, Zhuqiang Zhang, Qiang Dong, Jun Xiong, Bing Zhu

**Affiliations:** 1 National Laboratory of Biomacromolecules, CAS Center for Excellence in Biomacromolecules, Institute of Biophysics, Chinese Academy of Sciences, Beijing, 100101 China; 2 College of Life Sciences, University of Chinese Academy of Sciences, Beijing, 100049 China

**Keywords:** H3K27 acetylation, Enhancer, H3.3, Gene transcription

## Abstract

H3K27ac is well recognized as a marker for active enhancers and a great indicator of enhancer activity. However, its functional impact on transcription has not been characterized. By substituting lysine 27 in histone variant H3.3 with arginine in mouse embryonic stem cells, we diminish the vast majority of H3K27ac at enhancers. However, the transcriptome is largely undisturbed in these mutant cells, likely because the other enhancer features remain largely unchanged, including chromatin accessibility, H3K4me1, and histone acetylation at other lysine residues. Our results clearly reveal that H3K27ac alone is not capable of functionally determining enhancer activity.

**Electronic supplementary material:**

**Supplementary information** accompanies this paper at 10.1186/s13059-020-01957-w.

## Background

Enhancers are critical regulatory elements that control spatial and temporal gene expression [1], and they are demarcated by distinct chromatin modifications [2]. In mammals, putative enhancers can be predicted by their local chromatin signatures, including H3K4me1 and H3K27ac [3–5]. Among these enhancer marks, H3K27ac is of particular interest because it distinguishes active enhancers from poised ones [6]. To date, a large body of literature has well established a role for H3K27ac in indicating enhancer activity. On the other hand, whether H3K27ac functionally determinates enhancer activity is less clear because it is difficult to selectively target H3K27ac at enhancers. Nevertheless, it has been reported that H3K27ac plays an active role in cell identity control [7].

Enrichment of histone variant H3.3 and high histone turnover are other enhancer features [8–14]. Although mammalian H3.3 differs from canonical H3.1 by only five amino acids, H3.3 is deposited into specific chromatin regions [8, 9, 15, 16] by distinct histone chaperones [9, 17, 18] and is enriched for active histone modifications such as H3K4me3 and H3K27ac [19–21]. H3.3 is not essential for viability in *Drosophila* [22, 23]. However, complete loss of H3.3 in mice is embryonic lethal at an early stage and even cellular lethal as a result of mitotic defects [24]. Replacing lysine 27 with a nonmodifiable residue in one of the two genes encoding H3.3 in *Drosophila* causes male lethality and infertility and reduced global H3K27ac levels [25]. A similar reduction in H3K27ac levels was also observed in mouse embryonic stem cells (ESCs) depleted of H3.3 [26]. Therefore, we reasoned that targeting enhancer H3K27ac might be achievable by mutating both H3.3-encoding genes (*H3f3a* and *H3f3b*) and producing a homogenous population of H3.3 lysine 27-to-arginine (K27R) mutant histones in mouse ESCs.

## Results and discussion

**Fig. 1:**
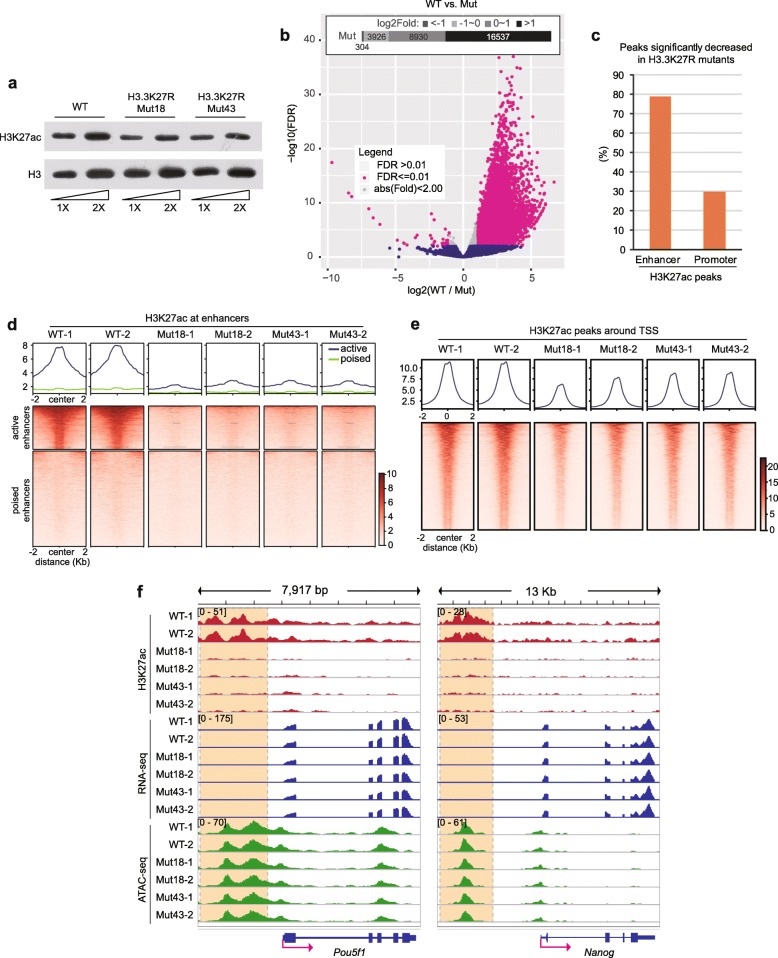
H3K27ac is dramatically decreased at enhancers in H3.3K27R mutant cells. **a** Western blot of H3K27ac from WT and H3.3K27R mutant mouse ESC lines. **b** Volcano plot illustrating the H3K27ac signal changes in H3.3K27R mutant ESCs in comparison with WT ESCs. Inlet diagram representing peak numbers in different categories of fold changes. FDR, false discovery rate. **c** Bar plot of the percentages of significantly decreased H3K27ac peaks at enhancers and promoters, respectively. **d**, **e** H3K27ac signals in WT and H3.3K27R mutant mouse ESC lines at enhancers (**d**) and TSSs (**e**). Upper, averaged profiles of the H3K27ac ChIP-seq signal at enhancers and TSSs, respectively. Lower, heatmap plots of the H3K27ac ChIP-seq signal at enhancers and TSSs, respectively. All biological duplicates are shown. **f** Genome browser representations of H3K27ac ChIP-seq, mRNA-seq, and ATAC-seq in WT and H3.3K27R mutant ESC lines at the *Pou5f1* and *Nanog* loci. Super-enhancer regions of each gene are highlighted with dashed line boxes

We utilized the CRISPR-Cas9 gene editing system to sequentially introduce mutations in both endogenous alleles of *H3f3a* and *H3f3b*, and the engineered mouse ESC clones were confirmed by Sanger sequencing (Additional file 2: Figure S1). We chose two H3.3K27R clones (Mut18 and Mut43) with similar H3.3 protein levels to those in wild-type (WT) ESCs for further investigations (Additional file 2: Figure S2a). The colony morphology and expression levels of key pluripotent genes were unchanged in H3.3K27R mutant cells compared with WT cells (Additional file 2: Figure S2b, S2c). We first measured the global H3K27ac level by Western blot analysis and observed a moderately reduced bulk H3K27ac level in mutant cells (Fig. 1a), which is expected because mouse ESCs contain much higher levels of canonical H3 histones, which remain possible to be acetylated at K27.

We then performed chromatin immunoprecipitation followed by deep sequencing (ChIP-seq) experiments in WT and the two H3.3K27R mutant lines, and all experiments were performed in biological duplicates. H3K27ac occupancy in the mutant lines displayed an extensive genome-wide loss (Additional file 2: Figure S3). We identified 13,037 H3K27ac peaks showing a more than twofold significant reduction in mutant cells, in contrast to only 40 H3K27ac peaks showing a more than twofold significant increase in mutant cells (Fig. 1b). We defined putative enhancers in WT ESCs with H3K4me1 ChIP-seq and observed that approximately 80% of enhancer-overlapping H3K27ac peaks exhibited significantly reduced H3K27ac in mutant lines using a twofold change cut-off, while the ratio was approximately 30% for H3K27ac peaks around promoters (Fig. 1c). These results indicate that we successfully targeted the enhancer H3K27ac level as we had planned. The different effects at enhancers and promoters suggest that H3.3 occupancy around active enhancers should be much higher than that at TSS regions. We analyzed recently published H3.3 ChIP-seq data [27] and indeed confirmed our prediction (Additional file 2: Figure S4).

We then divided all enhancers into active and poised groups according to the H3K27ac signal in WT cells. Strikingly, H3K27ac was almost completely depleted at active enhancers in H3.3K27R mutant ESCs (Fig. 1d). These results suggest that the vast majority of H3K27ac at active enhancers is on H3.3 and not on H3.1 or H3.2. This is consistent with high nucleosome turnover [13, 14] and high H3.3 occupancy ([27], Additional file 2: Figure S4) at enhancers. In contrast, H3K27ac signals around gene transcription start sites (TSSs) were moderately decreased in the mutants (Fig. 1e). H3.3 has been reported to promote the establishment of H3K27me3 at the bivalent promoters of developmentally regulated genes [28]. We mapped the distribution of H3K27me3 by ChIP-seq in WT and H3.3K27R mutant ESC lines and observed that H3K27me3 signals were moderately declined at bivalent regions (Additional file 2: Figure S5a), which could be attributed to the incorporation of the nonmodifiable H3.3K27R mutant histone. On the other hand, the genome-wide distribution of H3K27me3 was mostly unchanged in response to the H3.3K27R mutation (Additional file 2: Figure S5b). We also specifically looked at the enhancers of important pluripotent genes, such as *Pou5f1* (encoding Oct4) and *Nanog*, and observed a substantial reduction in H3K27ac (Fig. 1f).

**Fig. 2:**
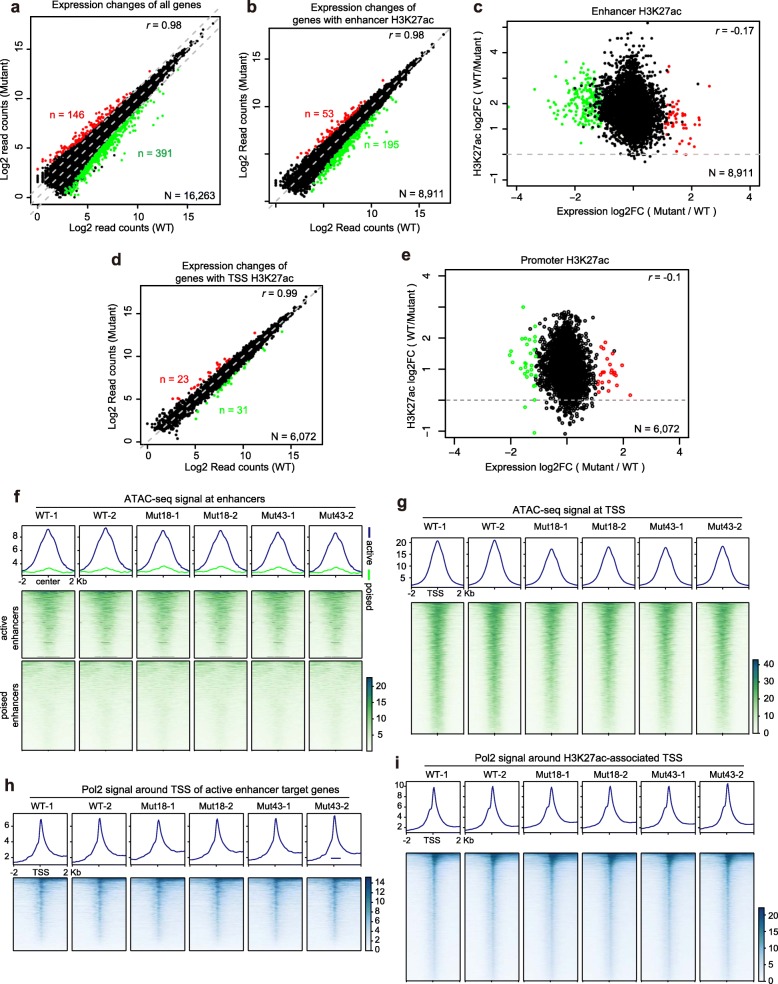
Transcriptome and enhancer identity are maintained in H3.3K27R mutant ESCs. **a** Dot plot depicting the transcriptional changes in all genes in H3.3K27R mutant ESCs compared to WT ESCs. Genes upregulated twofold with an FDR < 0.05 are shown in red. Genes downregulated twofold with an FDR < 0.05 are shown in green. **b** Dot plot depicting the transcriptional changes in genes associated with H3K27ac-occupied enhancers in H3.3K27R mutant ESCs compared to WT ESCs. **c** Dot plot revealing the relationship between transcriptional changes in genes associated with H3K27ac-occupied enhancers and H3K27ac ChIP-seq signal changes in H3.3K27R mutant ESCs compared to WT ESCs. **d** Dot plot depicting the transcriptional changes in genes associated with H3K27ac at TSSs in H3.3K27R mutant ESCs compared to WT ESCs. **e** Dot plot revealing the relationship between transcriptional changes in genes associated with H3K27ac at TSSs and H3K27ac ChIP-seq signal changes in H3.3K27R mutant ESCs compared to WT ESCs. **f**, **g** ATAC-seq signals in WT and H3.3K27R mutant mouse ESC lines at enhancers (**f**) and H3K27ac-occupied TSSs (**g**). Upper, averaged profiles of ATAC-seq signals at enhancers and H3K27ac-occupied TSSs, respectively. Lower, heatmap plots of ATAC-seq signals at enhancers and H3K27ac-occupied TSSs, respectively. All biological duplicates are shown. **h, i** RNA Pol II signals in WT and H3.3K27R mutant ESC lines around the TSSs of genes associated with distal active enhancers (**h**) and around TSSs overlapped with H3K27ac peaks (**i**). Upper, averaged profiles of the RNA Pol II signals in each group. Lower, heatmap plots of the RNA Pol II signals in each group

To evaluate the functional impact of the H3.3K27R mutation on transcription, we performed mRNA-seq in WT and the two mutant lines in biological duplicates. Despite the global reduction in H3K27ac in H3.3K27R mutant ESCs, the transcriptome change was minimal; in fact, the *r* value was 0.98 between the WT and mutant cells (Fig. 2a). Although 146 and 391 genes were upregulated or downregulated in H3.3K27R mutant ESCs, respectively (Fig. 2a), and the changes were highly consistent between the two individual H3.3K27R mutant ESC lines (Additional file 2: Figure S6), no obvious relationship was observed between H3K27ac and transcription change (see below). To define the target genes of active enhancers, we applied an activity-by-contact model to predict enhancer-gene connections in the WT mouse ESCs [29]. We associated 8911 genes with H3K27ac-marked active enhancers and observed only 53 upregulated and 195 downregulated genes in H3.3K27R mutant ESCs (Fig. 2b). Moreover, H3K27ac at enhancers of both the upregulated and downregulated genes was decreased (Fig. 2c). Similar events were observed for H3K27ac peaks around TSSs and their associated genes (Fig. 2d, e). Our results indicate that gene transcription is not sensitive to the dramatic decrease in H3K27ac at gene enhancers. Moreover, even though H3K27ac was greatly reduced at the enhancer regions of *Pou5f1* and *Nanog*, no transcriptional changes were observed in the mutant ESCs (Fig. 1f).

The activity-by-contact model outperformed other methods in predicting enhancer-gene connections [29], but the predicted associations are still not unequivocal. Super enhancers encompass large chromatin domains and harbor enormous amounts of H3K27ac and are associated with pluripotency genes in mouse ESCs [30]. The associations between super enhancers and their target genes are much more clearly defined in mouse ESCs. Therefore, we specifically examined super enhancer-associated genes, although H3K27ac levels were dramatically decreased at the super enhancers, few transcriptional changes occurred (Additional file 2: Figure S7). Only two genes, *Lefty2* and *Fam25c*, exhibited mild upregulation, and none of the genes exhibited significant downregulation (Additional file 2: Figure S7b, S7c). These results again indicate that H3K27ac is not functionally required for transcription, even for genes associated with super enhancers, in mouse ESCs.

To offer an explanation, we analyzed other features of enhancers in H3.3K27R mutant ESCs. H3K4me1 showed a mild decrease at enhancers and around H3K27ac-enriched TSSs (Additional file 2: Figure S8). Chromatin accessibility as determined by the amount of transposase-accessible chromatin with high-throughput sequencing (ATAC-seq) revealed that the open state of enhancers and TSSs remained largely unchanged in H3.3K27R mutant ESCs (Fig. 2f, g), suggesting that enhancer identities in mouse ESCs are well maintained. Similarly, H3K4me3 was well maintained at H3K27ac-occupied TSSs in H3.3K27R mutant ESCs (Additional file 2: Figure S9). Multiple lysine residues can be acetylated, and these acetylation events, such as H3K9ac, H3K18ac, H3K23ac, H3K122ac, and histone H4 acetylation, tend to be coenriched. ChIP-seq profiling of H3K9ac, H3K18ac, H3K122ac, H4K5ac, and H4K8ac in WT and H3.3K27R mutant ESCs showed that these acetylation events were generally unchanged or only mildly affected at enhancers and TSSs (Additional file 2: Figure S10). Lysine acetylation events neutralize positive charges on histone tails, attenuate the interaction between histone tails and DNA, and lead to chromatin opening [31]. H3K27ac is catalyzed by cyclic AMP response element-binding protein (CBP) and its paralogous protein P300, which also catalyze acetylation at many other histone lysine residues [32–34]. Therefore, a synergistic action by multiple acetylation events at these different residues is much more crucial for transcription than acetylation at a solo site, such as H3K27. Consistently, RNA Pol II occupancy was unaltered around the TSSs (Fig. 2h, i), reflecting globally undisturbed transcription initiation.

Among multiple histone lysine acetylation sites, H3K27ac is specifically utilized as a marker for active enhancers. Using chromatin accessibility as a proxy for promoter and enhancer activity, we analyzed the correlations between each individual acetylation ChIP-seq and ATAC-seq signals at promoters and enhancers. Pearson correlation revealed that indeed H3K27ac is the best marker associated with active enhancer, but acetylation events at other lysine residues also display positive correlations with enhancer accessibility (Additional file 2: Figure S11).

## Conclusions

Our results clearly indicate that the depletion of H3K27ac does not affect enhancer activity in mouse ESCs, whether it may have a stronger impact in other cells types such as somatic cells and cancer cells can be addressed using the same approach in future.

H3K27ac is and remains to be the best marker for enhancer indication. However, the depletion of H3K27ac at enhancer regions does not affect chromatin accessibility, gene transcription, and self-renewal of mouse ESCs. Therefore, maintenance of enhancer activity does not solely depend on H3K27ac; instead, H3K27ac has to work in concert with acetylation events on other histone lysine residues.

## Methods

### Cell culture and editing

J1 mouse ES cells [35] were cultured in standard ES medium in the presence of feeder cells. Prior to harvesting, ESCs were cultured under the feeder-free condition for two passages to remove feeder cells.

Gene editing was performed with CRISPR-Cas9 gene editing technology. Each gene was targeted by two sgRNAs: *H3f3a*: 5′-GCTTAATTAGCGCTCGACAC-3′, 5′-TACTAGTTGACTATACTAGA-3′; *H3f3b*: 5′-TGGTGGCCAGCTGTTTGCGG-3′, 5′-AAGCGCGCCCTCTACCGGCG-3′. Detailed strategies are presented in Additional file 1.

### ChIP-seq

ChIP experiments were performed as described previously [36] with minor modifications. In brief, cells were cross-linked with 1% formaldehyde for 9 min at room temperature. For RNA Pol II ChIP, cells were pre-cross-linked with 0.2 mM disuccinimidyl glutarate (DSG; ProteoChem, c1104) for 30 min at room temperature. Chromatin shearing was assisted with additional 0.1% sodium deoxycholate (0.5% for dual cross-linked samples). Adequate antibodies as indicated in the manufacturers’ instructions were used for precipitations. Library construction for deep sequencing was performed with KAPA Hyper Prep Kit (KAPA, KK8504) according to the manufacturers’ instructions.

### Antibodies

Antibodies against histone H3 (Abcam, ab1791), H3.3 (Millipore, 09-838), H3K4me1 (Active Motif, 39297), H3K4me3 (Millipore, 07-473), H3K9ac (Abcam, ab4441), H3K18ac (Active Motif, 39755), H3K27ac (Active Motif, 39133), H3K27me3 (Cell Signaling, 9733S), H3K122ac (Abcam, ab33309), histone H4 (ABclonal, A1131), H4K5ac (Active Motif, 39699), H4K8ac (Active Motif, 61103), and RNA Pol II (Active Motif, 39097) were obtained commercially.

### Sequencing and bioinformatic analysis

ChIP-seq and ATAC-seq libraries were sequenced (PE150) with Illumina NovaSeq 6000 (see Additional file 3: Table S1). mRNA-seq libraries were sequenced with the MGIDEQ-2000 (PE150) (see Additional file 3: Table S3). Mouse genome sequences (mm10) and *Drosophila* genome sequences (dm6) were concatenated to be used as the reference genome. H3.3 ChIP-seq data were downloaded from GEO database under the accession number of GSE117035 [27]. Please see Additional file 1 for the detailed data analysis strategies.

## Supplementary information

Additional file 1. Methods and supplementary references. (PDF 121 kb)

Additional file 2: **Figure S1.** Strategy for CRISPR-Cas9-mediated gene editing at *H3f3a* and *H3f3b* loci. **Figure S2.** H3.3K27R mutation does not affect the H3.3 protein level and pluripotent state of ESCs. **Figure S3.** Genome-wide decrease in H3K27ac levels in individual H3.3K27R mutant mouse ESC lines. **Figure S4.** Averaged profiles of H3.3 at active enhancers and H3K27-occupied promoters in mouse ESCs. **Figure S5.** H3.3K27R mutation does not affect the genome-wide distribution of H3K27me3 in mouse ESCs. **Figure S6.** Heatmap clustering of differentially expressed genes in WT and H3.3K27R mutant mouse ESC lines. **Figure S7.** H3.3K27R mutation does not affect the transcription of genes associated with super enhancers in mouse ESCs. **Figure S8.** H3K4me1 distribution at enhancers and H3K27ac-occupied promoters in WT and H3.3K27R mutant ESC lines. **Figure S9.** H3K4me3 distribution at H3K27ac-occupied promoters in WT and H3.3K27R mutant ESC lines. **Figure S10.** Averaged profiles of H3K9ac, H3K18ac, H3K122ac, H4K5ac, and H4K8ac at enhancers and H3K27ac-occupied promoters in WT and H3.3K27R mutant ESC lines. **Figure S11.** Barplots show Pearson correlation coefficients between ChIP-seq signal of histone acetylation and chromatin accessibility (ATAC-seq signal). (PDF 2893 kb)

Additional file 3: **Table S1.** Sequencing depth and mapping efficiency of ChIP-seq and ATAC-seq experiments. **Table S2.** Peak numbers called in ATAC-seq and ChIP-seq experiments. **Table S3.** Stats for mRNA-seq experiments. Table S4 Sequences of DNA oligos used in this study. (PDF 459 kb)

Additional file 4. Review history.

## References

[1] Serfling E, Jasin M, Schaffner W (1985). Enhancers and eukaryotic gene transcription. Trends Genet.

[2] Calo E, Wysocka J (2013). Modification of enhancer chromatin: what, how, and why?. Mol Cell.

[3] Heintzman ND, Stuart RK, Hon G, Fu Y, Ching CW, Hawkins RD (2007). Distinct and predictive chromatin signatures of transcriptional promoters and enhancers in the human genome. Nat Genet.

[4] Heintzman ND, Hon GC, Hawkins RD, Kheradpour P, Stark A, Harp LF (2009). Histone modifications at human enhancers reflect global cell-type-specific gene expression. Nature..

[5] Wang Z, Zang C, Rosenfeld JA, Schones DE, Barski A, Cuddapah S (2008). Combinatorial patterns of histone acetylations and methylations in the human genome. Nat Genet.

[6] Creyghton MP, Cheng AW, Welstead GG, Kooistra T, Carey BW, Steine EJ (2010). Histone H3K27ac separates active from poised enhancers and predicts developmental state. Proc Natl Acad Sci U S A.

[7] Lavarone E, Barbieri CM, Pasini D (2019). Dissecting the role of H3K27 acetylation and methylation in PRC2 mediated control of cellular identity. Nat Commun.

[8] Mito Y, Henikoff JG, Henikoff S (2005). Genome-scale profiling of histone H3.3 replacement patterns. Nat Genet.

[9] Goldberg AD, Banaszynski LA, Noh K-M, Lewis PW, Elsaesser SJ, Stadler S (2010). Distinct factors control histone variant H3.3 localization at specific genomic regions. Cell..

[10] Chen P, Zhao J, Wang Y, Wang M, Long H, Liang D (2013). H3.3 actively marks enhancers and primes gene transcription via opening higher-ordered chromatin. Genes Dev.

[11] Huang C, Zhang Z, Xu M, Li Y, Li Z, Ma Y (2013). H3.3-H4 tetramer splitting events feature cell-type specific enhancers. PLoS Genet.

[12] Huang C, Zhu B (2014). H3.3 turnover: a mechanism to poise chromatin for transcription, or a response to open chromatin?. Bioessays..

[13] Kraushaar DC, Jin W, Maunakea A, Abraham B, Ha M, Zhao K (2013). Genome-wide incorporation dynamics reveal distinct categories of turnover for the histone variant H3.3. Genome Biol.

[14] Deaton AM, Gomez-Rodriguez M, Mieczkowski J, Tolstorukov MY, Kundu S, Sadreyev RI et al. Enhancer regions show high histone H3.3 turnover that changes during differentiation. eLife. 2016;5:e15316. 10.7554/eLife.15316 PMC4965263 27304074

[15] Chow Cheok‐Man, Georgiou Andrew, Szutorisz Henrietta, Maia e Silva Alexandra, Pombo Ana, Barahona Isabel, Dargelos Elise, Canzonetta Claudia, Dillon Niall (2005). Variant histone H3.3 marks promoters of transcriptionally active genes during mammalian cell division. EMBO reports.

[16] Dunleavy EM, Almouzni G, Karpen GH (2011). H3.3 is deposited at centromeres in S phase as a placeholder for newly assembled CENP-A in G(1) phase. Nucleus.

[17] Tagami H, Ray-Gallet D, Almouzni G, Nakatani Y (2004). Histone H3.1 and H3.3 complexes mediate nucleosome assembly pathways dependent or independent of DNA synthesis. Cell..

[18] Ray-Gallet D, Woolfe A, Vassias I, Pellentz C, Lacoste N, Puri A (2011). Dynamics of histone H3 deposition in vivo reveal a nucleosome gap-filling mechanism for H3.3 to maintain chromatin integrity. Mol Cell.

[19] McKittrick E, Gafken PR, Ahmad K, Henikoff S (2004). Histone H3.3 is enriched in covalent modifications associated with active chromatin. Proc Natl Acad Sci USA.

[20] Hake SB, Garcia BA, Duncan EM, Kauer M, Dellaire G, Shabanowitz J (2006). Expression patterns and post-translational modifications associated with mammalian histone H3 variants. J Biol Chem.

[21] Loyola A, Bonaldi T, Roche D, Imhof A, Almouzni G (2006). PTMs on H3 variants before chromatin assembly potentiate their final epigenetic state. Mol Cell.

[22] Hodl M, Basler K (2009). Transcription in the absence of histone H3.3. Curr Biol.

[23] Sakai A, Schwartz BE, Goldstein S, Ahmad K (2009). Transcriptional and developmental functions of the H3.3 histone variant in Drosophila. Curr Biol.

[24] Jang CW, Shibata Y, Starmer J, Yee D, Magnuson T (2015). Histone H3.3 maintains genome integrity during mammalian development. Genes Dev.

[25] Leatham-Jensen M, Uyehara CM, Strahl BD, Matera AG, Duronio RJ, McKay DJ (2019). Lysine 27 of replication-independent histone H3.3 is required for Polycomb target gene silencing but not for gene activation. PLoS Genet.

[26] Martire S, Gogate AA, Whitmill A, Tafessu A, Nguyen J, Teng YC (2019). Phosphorylation of histone H3.3 at serine 31 promotes p300 activity and enhancer acetylation. Nat Genet.

[27] Wang Y, Long H, Yu J, Dong L, Wassef M, Zhuo B (2018). Histone variants H2A.Z and H3.3 coordinately regulate PRC2-dependent H3K27me3 deposition and gene expression regulation in mES cells. BMC Biol.

[28] Banaszynski LA, Wen D, Dewell S, Whitcomb SJ, Lin M, Diaz N (2013). Hira-dependent histone H3.3 deposition facilitates PRC2 recruitment at developmental loci in ES cells. Cell..

[29] Fulco CP, Nasser J, Jones TR, Munson G, Bergman DT, Subramanian V (2019). Activity-by-contact model of enhancer-promoter regulation from thousands of CRISPR perturbations. Nat Genet.

[30] Whyte WA, Orlando DA, Hnisz D, Abraham BJ, Lin CY, Kagey MH (2013). Master transcription factors and mediator establish super-enhancers at key cell identity genes. Cell..

[31] Eberharter A, Becker PB (2002). Histone acetylation: a switch between repressive and permissive chromatin. Second in review series on chromatin dynamics. EMBO Rep.

[32] Bannister AJ, Kouzarides T (1996). The CBP co-activator is a histone acetyltransferase. Nature..

[33] Ogryzko VV, Schiltz RL, Russanova V, Howard BH, Nakatani Y (1996). The transcriptional Coactivators p300 and CBP are histone Acetyltransferases. Cell..

[34] Sen P, Lan Y, Li CY, Sidoli S, Donahue G, Dou Z (2019). Histone acetyltransferase p300 induces de novo super-enhancers to drive cellular senescence. Mol Cell.

[35] Yin Y, Yan P, Lu J, Song G, Zhu Y, Li Z (2015). Opposing roles for the lncRNA haunt and its genomic locus in regulating HOXA gene activation during embryonic stem cell differentiation. Cell Stem Cell.

[36] Xiong J, Zhang Z, Chen J, Huang H, Xu Y, Ding X (2016). Cooperative action between SALL4A and TET proteins in stepwise oxidation of 5-methylcytosine. Mol Cell.

[37] Zhang T, Zhang Z, Dong Q, Xiong J, Zhu B. The role of histone H3K27 acetylation for enhancer activity in embryonic stem cells. GSE141525. Gene Expression Omnibus. https://www.ncbi.nlm.nih.gov/geo/query/acc.cgi?acc=GSE141525 (2020). Accessed 9 Feb 2020.

