# Supplementary material for: Histone H3K27 acetylation is dispensable for enhancer activity in mouse embryonic stem cells
Source: Genome Biol. 2020 Feb 21;21:45. doi: 10.1186/s13059-020-01957-w (PMC7035716; doi:10.1186/s13059-020-01957-w)
Supplement: Supplementary file 1 — Methods and supplementary references. (PDF 121 kb) [file 13059_2020_1957_MOESM1_ESM.pdf]

Methods

Cell culture

J1 mouse ESCs [1] were cultured on 0.1% gelatin (Millipore, ES-006-B)-coated feeder cell plates. The medium contained Dulbecco's Modified Eagle Medium (DMEM, Life Technologies, 11995), 15% fetal bovine serum (FBS, Hyclone, SH30070.03), 2 mM L-Glutamine (BBI Life Sciences, E607004-0100), 0.1 mM non-essential amino acids (Sigma, M7145), 0.1 mM ß-mercaptoethanol (Gibco, 21985023), 1,000 U/ml leukemia inhibitory factor (LIF, Millipore, ESG1107), and 1× Penicillin-Streptomycin (BBI Life Sciences, E607011-0100). Cultures were incubated at 37 ˚C with 5% CO_2 in a humidified incubator.

Prior to all experiments, feeder cells were depleted first as following. Trypsinized cells were incubated 37 ˚C with 5% CO_2 in a humidified incubator for 30 min to let feeder cells adhere to the culture plate. ESCs in suspension were transferred to 0.1% gelatin-coated plates and cultured for two passages without feeder cells.

Generation of H3.3K27R point mutation cell lines

To generate lysine-to-arginine substitution at endogenous *H3f3a* and *H3f3b* loci, we used CRISPR-Cas9 gene editing technology in J1 mouse ESCs (Additional file 2: Figure S1). sgRNA design was based on website http://crispor.tefor.net/crispor.py. Each gene was targeted by two sgRNAs around (*H3f3a*) or at (*H3f3b*) the K27-encoding exon and the targeting sequences were listed: *H3f3a*: 5'-GCTTAATTAGCGCTCGACAC-3', 5'-TACTAGTTGACTATACTAGA-3'; *H3f3b*: 5'-TGGTGGCCAGCTGTTTGCGG-3', 5'-AAGCGCGCCCTCTACCGGCG-3'. sgRNAs were cloned into lentiCRISPRv2 (Addgene, 52961) [2], and cleavage efficiency was confirmed by Surveyor Mutation Detection Kits (IDT, 706020).

To construct donor plasmids for homologous recombination-mediated nucleotide substitution, a 2.5-kb region spanning the mutation site of mouse *H3f3a* gene and a 1.7-kb region spanning the mutation site of *H3f3b* gene were cloned, respectively, from the genomic DNA of mouse ESCs, and inserted into pEASY-Blunt Zero plasmid (Transgen Biotech) by Seamless Cloning Kit (Beyotime Biotechnology, D7010M). Lysine-to-arginine substitution was achieved by mutagenesis PCR (TOYOBO, KOD-201) to exchange the corresponding amino acid codons. PAM sites of the sgRNA targeting sequence in donor plasmids were mutated to avoid undesired cutting on donor plasmids.

To mutate both alleles of *H3f3a* gene by a single transfection, a PGK-driven mCherry expression cassette or a PGK-driven eGFP expression cassette flanked by *LoxP* sequences at both sides was inserted into the cloned intronic region of the H3f3a^{K27R} donor plasmid, respectively. After co-transfected with 2 sgRNA plasmids into mouse ESCs by Lipofectamine LTX (ThermoFisher, 15338100), a puromycin selection (1 μg/ml) was performed for 24 hours to enrich Cas9 and sgRNA expressed cells. Flow cytometry was carried out seven days after drug selection to sort mCherry and eGFP double-positive cells. Then the cells were transfected with a plasmid expressing Cre enzyme to remove the PGK-mCherry and eGFP cassette. After recovery for 3-4 days, mCherry and eGFP double-negative cells were sorted by flow cytometry and seeded into pre-coated 96-well plates by limited dilution. Individual clones were examined by genotyping PCR and clones with homozygous mutation were kept and validated by both DNA sequencing and cDNA sequencing.

For *H3f3b* gene, homologous recombination donor carrying the lysine 27-to-arginine substitution was co-transfected with two sgRNAs. Thirty-six hours after transfection, a puromycin selection (1 μg/ml) was performed for 24 hours to enrich Cas9 and sgRNA expressed cells. After recovery for

3-4 days, cells were seeded into pre-coated 96-well plates by limited dilution. Genotyping and sequencing validation was performed to select clones with homozygous mutation. The expression level of mutated H3.3A and H3.3B from their endogenous loci was evaluated by Western analysis. Sequences of DNA oligos used in this study were listed in Additional file 3: Table S4.

Total RNA extraction, cDNA synthesis, and mRNA-seq

Cells grown in a 3.5 cm dish was extracted with 1 ml of TRIzol™ reagent (ThermoFisher, 15596026) according to the manufacturer's recommendations. One microgram of total RNA was reverse-transcribed into cDNA using All-in One cDNA Synthesis SuperMix (Bimake, B24403) according to the manufacturer's recommendations. cDNA of *H3f3a* and *H3f3b* was amplified with 2× Rapid Taq Master Mix (Vazyme, P222-AA) and sent for Sanger sequencing to validate the correctly mutated ESC clones.

For mRNA-seq, total RNA was prepared with two biological replicates for each cell line. 2 µg of *Drosophila* total RNA was added to each sample (20 µg) as a spike-in at the ratio of 1:10. Poly-A tailed mRNA molecules were captured using attached oligo-dT and subjected to library construction according to the manufacturer's recommendations.

Histone extraction

Cells cultured in 3.5 cm dishes were trypsinized and washed once with PBS. Cell pellets were resuspended with 600 µl of 0.2 N HCl and incubated at 4 ˚C with rotation for more than 3 hours. The lysates were cleared by centrifugation at 12,000 rpm for 10 min at 4 ˚C and transferred to new tubes. Acid-extracted histones were precipitated by adding 300 µl of trichloroacetic acid solution (SIGMA, T0699) and incubating on ice for 30 min, and then pelleting by centrifuging at 12,000

rpm for 10 min at 4 ˚C. The white pellet was washed three times with ice-cold acetone, air dried in a fume cupboard, and resuspended with 100 µl of water. Histone samples were stored at -20 ˚C.

ATAC-seq

ATAC-seq was performed as previously described [3] with some modifications. Briefly, 50,000 cells were washed once with 50 µl of ice-cold PBS, then lysed with 50 µl of ice-cold lysis buffer (10 mM Tris-HCl, pH 7.5, 10 mM NaCl, 3 mM $MgCl_2$, 0.1% NP-40, 0.1% Tween-20, and 0.01% Digitonin) by incubating on ice for 10 min. The pellet was washed once with 1 ml of washing buffer (10 mM Tris-HCl, pH 7.5, 10 mM NaCl, 3 mM $MgCl_2$, and 0.1% Tween-20). Transposition reaction was performed in 50 µl of reaction mix containing 10 µl of 5× TTBL (Vazyme, TD501), 5 µl of TTE Mix V50 (Vazyme, TD501), 16.5 µl of PBS, 0.5 µl of 10% Tween-20, 0.5 µl of 1% digitonin, and 17.5 µl of PCR-grade water. The mixture was incubated at 37 ˚C for 30 min on a thermomixer with shaking at 1,000 rpm. Then, DNA was extracted by phenol-chloroform and amplified by KAPA HiFi HotStart ReadyMix (KAPA Biosystems, KK2611) with barcoded primers (Vazyme, TD202). DNA fragments ranging from 200 bp to 1,000 bp were selected for deep sequencing.

Data analysis

Reads mapping

ChIP-seq and ATAC-seq libraries were sequenced (PE150) on Illumina NovaSeq 6000 platform by Berry Genomics Beijing Co. Ltd (see Additional file 3: Table S1). WT mouse ESC line and two H3.3K27R mutant lines were sequenced in two independent biological replicates, respectively. Sequencing qualities were evaluated with FastQC (v0.11.8) software and then pre-processed using

‘trim_galore’ (v0.6.2) to filter low quality bases or adaptor contaminations. Trimmed sequences were aligned to mouse genome assembly (GRCm38/mm10) using Bowtie2 [4] (v2.3.5) with a maximum of 2 mismatches allowed. Only the uniquely mapped reads were kept, and duplicate reads were removed with Samtools (v1.9).

Peak calling

Peaks were called using MACS2 [5] for H3K27ac, H3K4me1, H3K4me3, and H3K27me3 modifications, in comparison to the corresponding input libraries. Q-value cutoffs were set empirically to get comparable peak numbers (see Additional file 3: Table S2). Common peaks between replicates of each modification were used for further analysis. Bivalent peaks were defined as common peaks between H3K27me3 and H3K4me3 peaks.

Differential analysis of H3K27ac peaks

Differential occupancy analysis of H3K27ac between WT and mutant lines were performed using R package DiffBind [6] v2.14.0, with *P*-value threshold 0.01 and 2-fold change cut-off. WT and Mut18 were firstly compared (each with two replicates), and then WT and Mut43. As two mutant lines coincided well, we combined these two lines of mutants into 4 replicates (2 reps of WT vs. 4 reps of Muts), and derived the final set of differentially binding sites.

Enhancer identification

To identify enhancers, H3K4me1 peaks that overlapped with H3K4me3 peaks were firstly excluded, and then H3K4me1 peaks within TSS ± 1 kb were excluded to keep distal H3K4me1 as enhancers. All putative enhancers were categorized into active and poised enhancers according to overlapping with H3K27ac peaks or not, respectively. Neighboring enhancers located within 500 bp were merged. Top 347 enhancers with the highest H3K27ac signals were defined as super enhancers (SE), according to the method described by Hnisz *et al*. [7]. All intersection calculations

were done using bedtools v2.28.0 software. Target genes of enhancers were defined using activity-by-contact (ABC) model described by CP. Fulco et al. [8], with which ABC_score threshold was set to 0.1 to get appropriate number of enhancer-gene connections. For the calculation of enhancer H3K27ac signals for target genes that possess more than one enhancer, signals of all its enhancers were averaged for each gene.

Heatmap and genomic profile

Genome profile files were generated with deepTools [9] with normalized library size of 10 million read pairs, and visualized with IGV genome browser [10]. TSS annotation data were obtained from "refGene" table of UCSC mm10 databases. Reads densities of specific gene sets or regions of interest were averaged based on "bigwig" signals generated from reads mapping files. This pipeline was applied to ChIP-seq and ATAC-seq data similarly.

mRNA-seq

mRNA-seq libraries were sequenced on the MGIDEQ-2000 platform (PE150) by MGI Tech, Shenzhen Co. Ltd (see Additional file 3: Table S3). Mouse genome sequences (mm10) and *Drosophila* genome sequences (dm6) were concatenated to be used as the reference genome. Using STAR aligner (v2.7.0f), mRNA-seq read pairs were mapped to the concatenated genome and then discriminated into two distinct species. To use the *Drosophila* cells as spike-ins, we used DESeq2 [11] software to estimate the "size factors" from reads of *Drosophila* transcriptome, and then applied it to mouse datasets, as described by Taruttis *et. al*. [12]. Differentially expressed genes (DEGs) were derived using the cutoff $\alpha <= 0.05$ and foldchange >= 2. As two mutant lines coincided well, we combined these two lines of mutants into 4 replicates (2 reps of WT vs. 4 reps of Muts), and derived the final set of differentially expressed genes.

Supplementary references

1. Yin Y, Yan P, Lu J, Song G, Zhu Y, Li Z *et al*. Opposing Roles for the lncRNA Haunt and Its Genomic Locus in Regulating HOXA Gene Activation during Embryonic Stem Cell Differentiation. Cell Stem Cell. 2015;16:504-16.
2. Sanjana NE, Shalem O, Zhang F. Improved vectors and genome-wide libraries for CRISPR screening. Nat Methods. 2014;11:783-4.
3. Corces MR, Trevino AE, Hamilton EG, Greenside PG, Sinnott-Armstrong NA, Vesuna S *et al*. An improved ATAC-seq protocol reduces background and enables interrogation of frozen tissues. Nat Methods. 2017;14:959-62.
4. Langmead B, Salzberg SL. Fast gapped-read alignment with Bowtie 2. Nat Methods. 2012;9:357-9.
5. Zhang Y, Liu T, Meyer CA, Eeckhoute J, Johnson DS, Bernstein BE *et al*. Model-based analysis of ChIP-Seq (MACS). Genome Biol. 2008;9:R137.
6. Ross-Innes CS, Stark R, Teschendorff AE, Holmes KA, Ali HR, Dunning MJ *et al*. Differential oestrogen receptor binding is associated with clinical outcome in breast cancer. Nature. 2012;481:389-93.
7. Hnisz D, Abraham BJ, Lee TI, Lau A, Saint-Andre V, Sigova AA *et al*. Super-enhancers in the control of cell identity and disease. Cell. 2013;155:934-47.
8. Fulco CP, Nasser J, Jones TR, Munson G, Bergman DT, Subramanian V *et al*. Activity-by-contact model of enhancer-promoter regulation from thousands of CRISPR perturbations. Nat Genet. 2019;51:1664-9.
9. Ramirez F, Ryan DP, Gruning B, Bhardwaj V, Kilpert F, Richter AS *et al*. deepTools2: a next generation web server for deep-sequencing data analysis. Nucleic Acids Res. 2016;44:W160-5.
10. Robinson JT, Thorvaldsdottir H, Winckler W, Guttman M, Lander ES, Getz G *et al*. Integrative genomics viewer. Nat Biotechnol. 2011;29:24-6.

11. Love MI, Huber W, Anders S. Moderated estimation of fold change and dispersion for RNA-seq data with DESeq2. Genome Biol. 2014;15:550.

12. Taruttis F, Feist M, Schwarzfischer P, Gronwald W, Kube D, Spang R *et al*. External calibration with Drosophila whole-cell spike-ins delivers absolute mRNA fold changes from human RNA-Seq and qPCR data. Biotechniques. 2017;62:53-61.
