# Supplementary material for: Histone H3K27 acetylation is dispensable for enhancer activity in mouse embryonic stem cells
Source: Genome Biol. 2020 Feb 21;21:45. doi: 10.1186/s13059-020-01957-w (PMC7035716; doi:10.1186/s13059-020-01957-w)
Supplement: Supplementary file 2 — Figure S1. Strategy for CRISPR-Cas9-mediated gene editing at H3f3a and H3f3b loci. Figure S2. H3.3K27R mutation does not affect the H3.3 protein level and pluripotent state of ESCs. Figure S3. Genome-wide decrease in H3K27ac levels in individual H3.3K27R mutant mouse ESC lines. Figure S4. Averaged profiles of H3.3 at active enhancers and H3K27-occupied promoters in mouse ESCs. Figure S5. H3.3K27R mutation does not affect the genome-wide distribution of H3K27me3 in mouse ESCs. Figure S6. Heatmap clustering of differentially expressed genes in WT and H3.3K27R mutant mouse ESC lines. Figure S7. H3.3K27R mutation does not affect the transcription of genes associated with super enhancers in mouse ESCs. Figure S8. H3K4me1 distribution at enhancers and H3K27ac-occupied promoters in WT and H3.3K27R mutant ESC lines. Figure S9. H3K4me3 distribution at H3K27ac-occupied promoters in WT and H3.3K27R mutant ESC lines. Figure S10. Averaged profiles of H3K9ac, H3K18ac, H3K122ac, H4K5ac, and H4K8ac at enhancers and H3K27ac-occupied promoters in WT and H3.3K27R mutant ESC lines. Figure S11. Barplots show Pearson correlation coefficients between ChIP-seq signal of histone acetylation and chromatin accessibility (ATAC-seq signal). (PDF 2893 kb) [file 13059_2020_1957_MOESM2_ESM.pdf]

Supplementary figures:

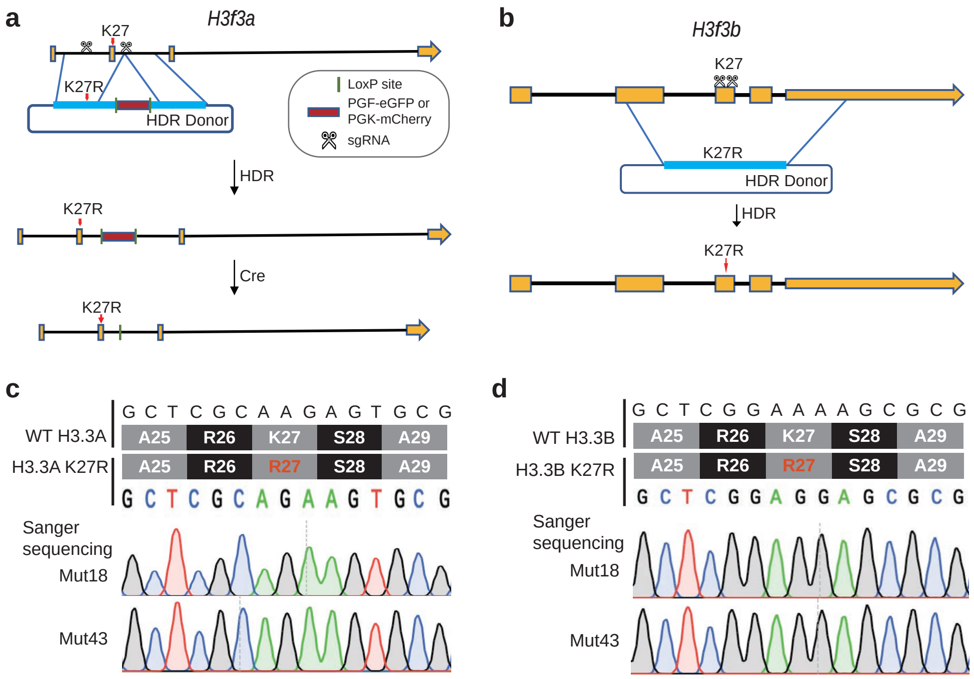

Figure S1. Strategy for CRISPR-Cas9-mediated gene editing at *H3f3a* and *H3f3b* loci. (**a-b**) Schematic representation of the CRISPR-Cas9-mediated nucleotide substitution at *H3f3a* and *H3f3b* loci in mouse ESCs. HDR, homology-directed repair. (**c-d**) Sanger sequencing of reverse transcribed mRNA sequences to validate successfully mutated ESC clones (Mut18 and Mut43).

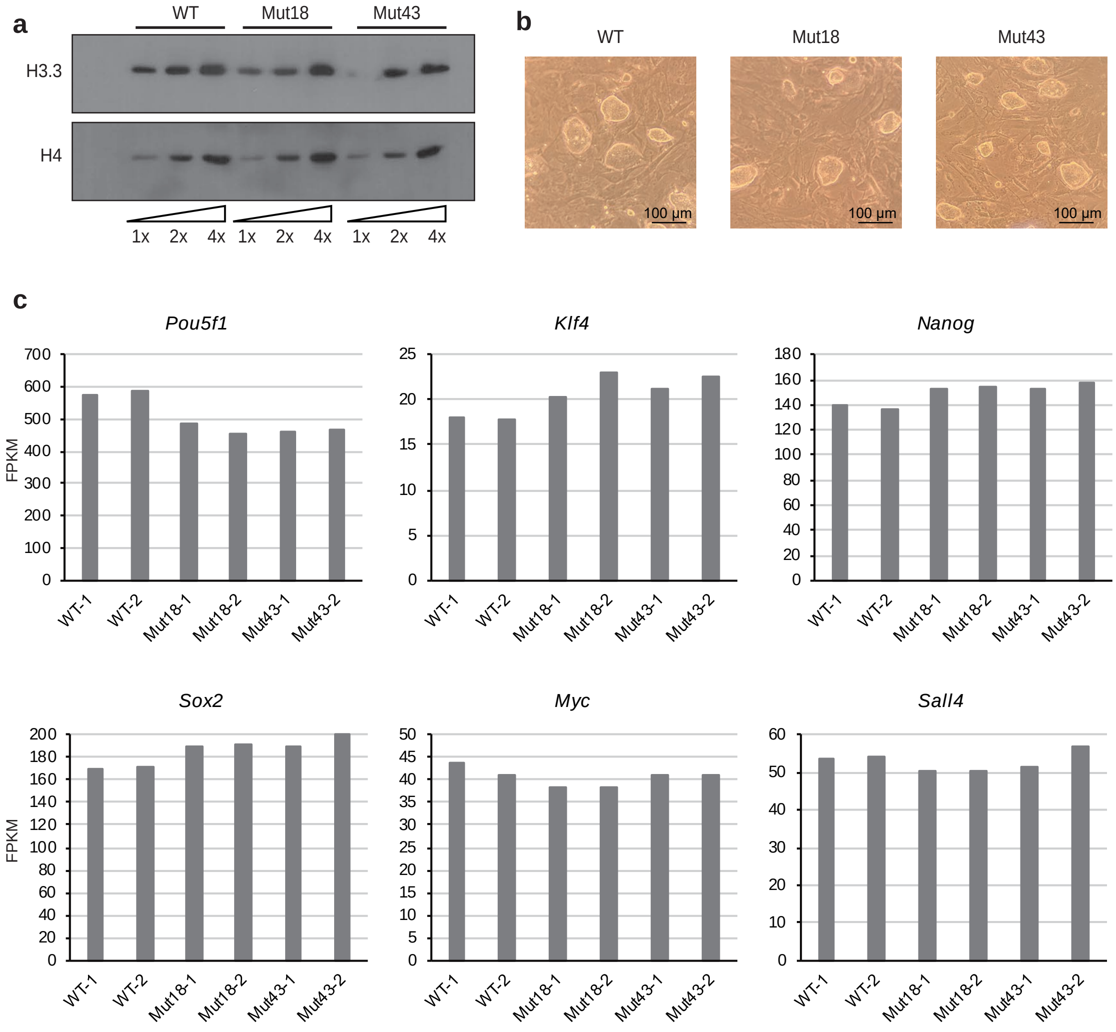

Figure S2. H3.3K27R mutation does not affect the H3.3 protein level and pluripotent state of ESCs. (**a**) Western blot of H3.3 protein from WT and H3.3K27R mutant mouse ESC lines. (**b**) Representative images showing the colony morphology of WT and H3.3K27R mutant mouse ESC lines. (**c**) Expression levels of key pluripotent genes in WT and H3.3K27R mutant mouse ESC lines.

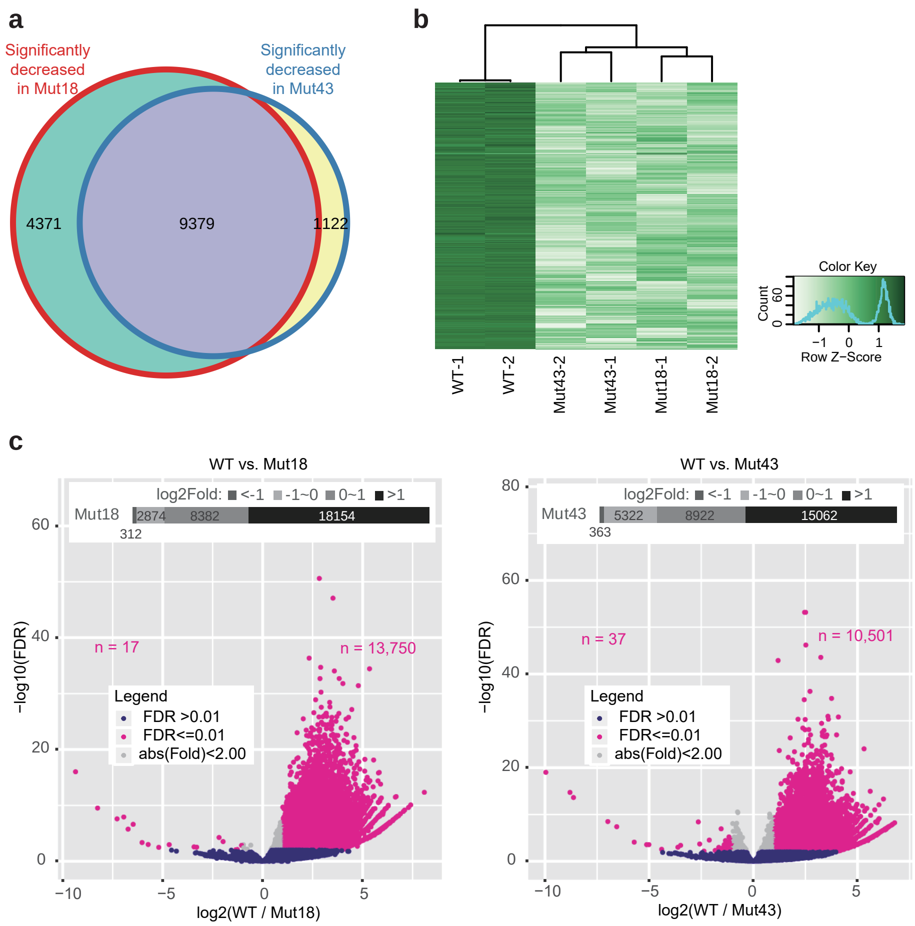

Figure S3. Genome-wide decrease in H3K27ac levels in individual H3.3K27R mutant mouse ESC lines. (**a**) Venn diagram showing comparable H3K27ac decreases in both H3.3K27R mutant mouse ESC lines. (**b**) Heatmap clustering of H3K27ac ChIP-seq signals showing a genome-wide reduction of H3K27ac in H3.3K27R mutant mouse ESC lines. Consensus peak set used are those peaks shared by at least two samples. (**c**) Volcano plots illustrating the H3K27ac ChIP-seq signal changes in two H3.3K27R mutant ESC lines in comparison with WT ESCs, respectively. Inlet diagram representing peak numbers in different categories of fold changes. FDR, false discovery rate.

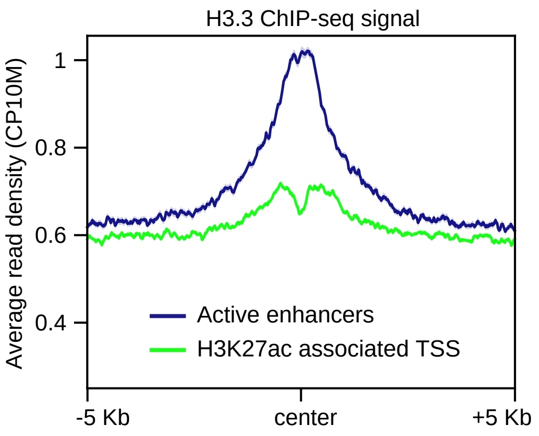

Figure S4. Averaged profiles of H3.3 at active enhancers and H3K27-occupied promoters in mouse ESCs. Reads densities were normalized to the sequencing depth of 10 M read pairs, and averaged for all covered regions of interest. H3.3 ChIP-seq data were adopt from GEO database under the accession number of GSE117035.

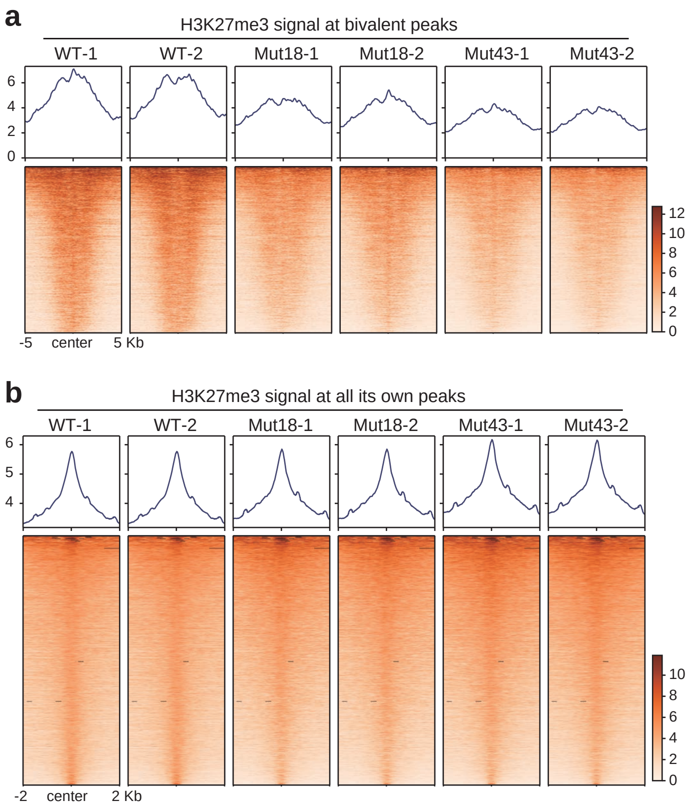

Figure S5. H3.3K27R mutation does not affect the genome-wide distribution of H3K27me3 in mouse ESCs. (**a**) Averaged profiles (upper) and heatmap plots (lower) of the H3K27me3 ChIP-seq signal at bivalent peaks in WT and H3.3K27R mutant mouse ESC lines. All biological duplicates are shown. (**b**) Averaged profiles (upper) and heatmap plots (lower) of the H3K27me3 ChIP-seq signal at all H3K27me3 peaks in WT and H3.3K27R mutant mouse ESC lines. All biological duplicates are shown.

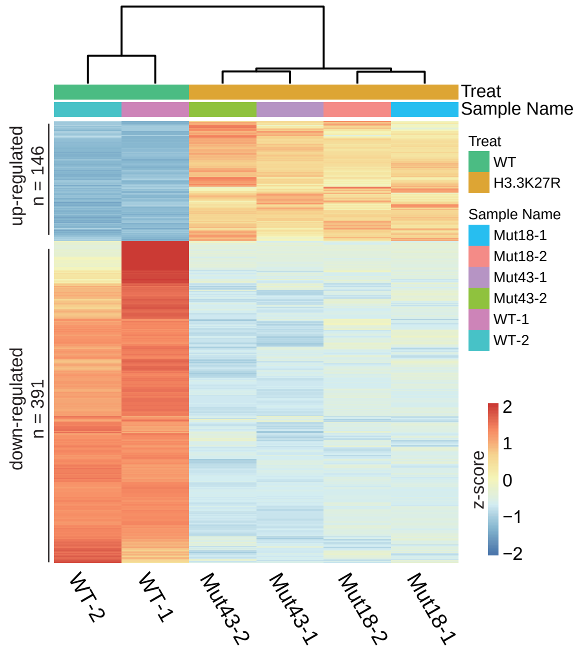

Figure S6. Heatmap clustering of differentially expressed genes in WT and H3.3K27R mutant mouse ESC lines.

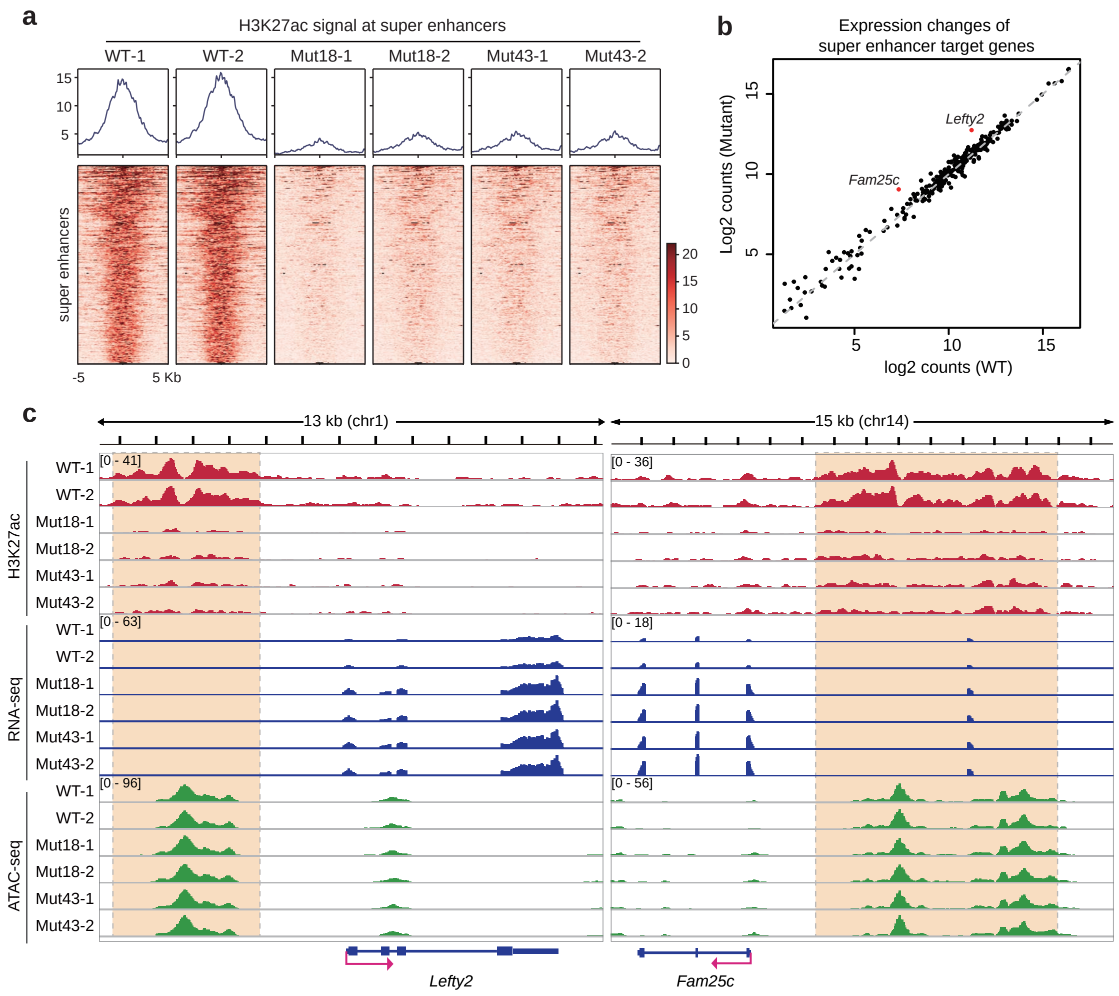

Figure S7. H3.3K27R mutation does not affect the transcription of genes associated with super enhancers in mouse ESCs. (**a**) Heatmap plots of the H3K27ac ChIP-seq signal at super-enhancers in WT and H3.3K27R mutant mouse ESC lines. All biological duplicates are shown. (**b**) Dot plot depicting transcription changes in genes associated with super enhancers in H3.3K27R mutant mouse ESCs compared to WT ESCs. Genes upregulated for 2-fold with an FDR < 0.05 are shown in red. (**c**) Genome browser representations of H3K27ac ChIP-seq, mRNA-seq, and ATAC-seq signal in WT and H3.3K27R mutant ESC lines at the *Lefty2* and *Fam25c* loci. Super-enhancer regions of each gene are highlighted with dashed line boxes.

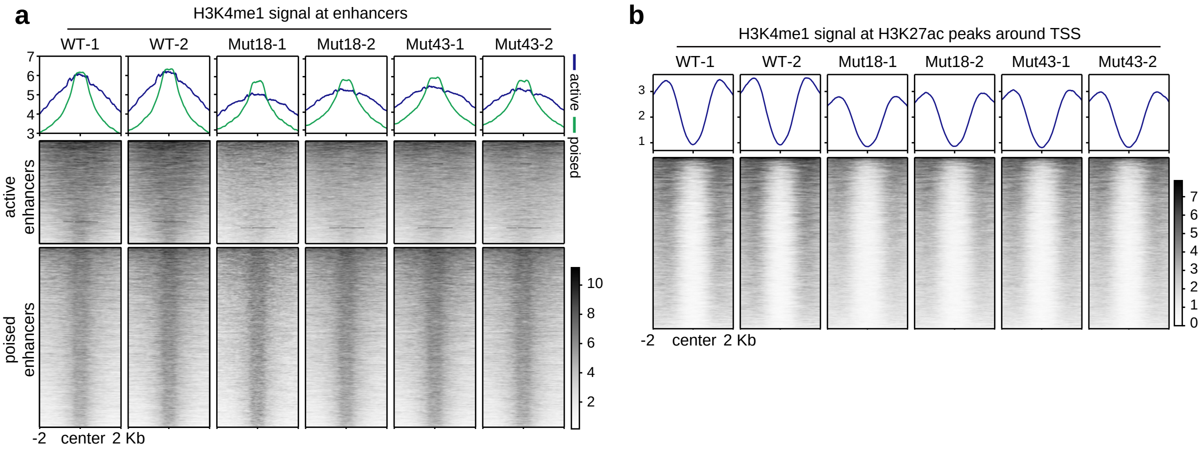

Figure S8. H3K4me1 distribution at enhancers and H3K27ac-occupied promoters in WT and H3.3K27R mutant ESC lines. (**a-b**) H3K4me1 ChIP-seq signals in WT and H3.3K27R mutant mouse ESC lines at enhancers (**a**) and H3K27ac-occupied TSS (**b**). Upper, averaged profiles of the H3K4me1 ChIP-seq signal at enhancers and H3K27ac-occupied TSS, respectively. Lower, heatmap plots of the H3K4me1 ChIP-seq signal at enhancers and H3K27ac-occupied TSS, respectively. All biological duplicates are shown.

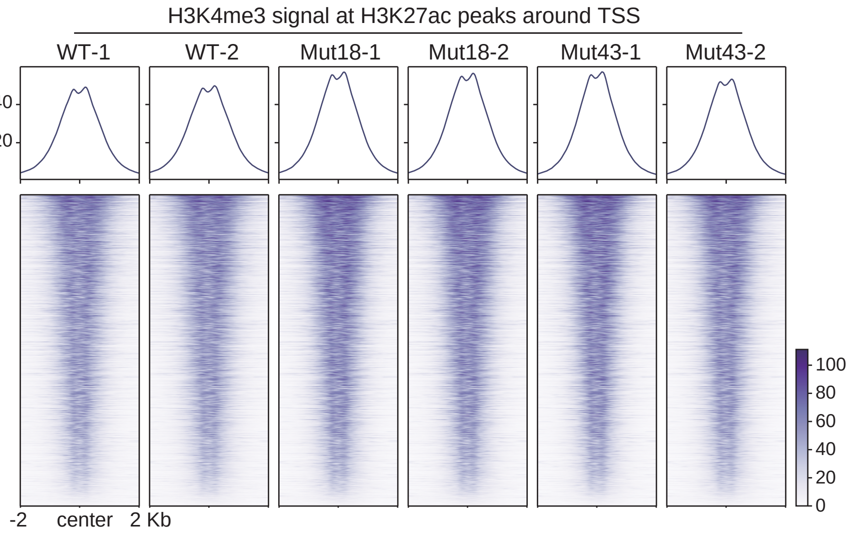

Figure S9. H3K4me3 distribution at H3K27ac-occupied promoters in WT and H3.3K27R mutant ESC lines. Upper, averaged profiles of the H3K4me3 ChIP-seq signal at H3K27ac-occupied TSS. Lower, heatmap plots of the H3K4me3 ChIP-seq signal at H3K27ac-occupied TSS. All biological duplicates are shown.

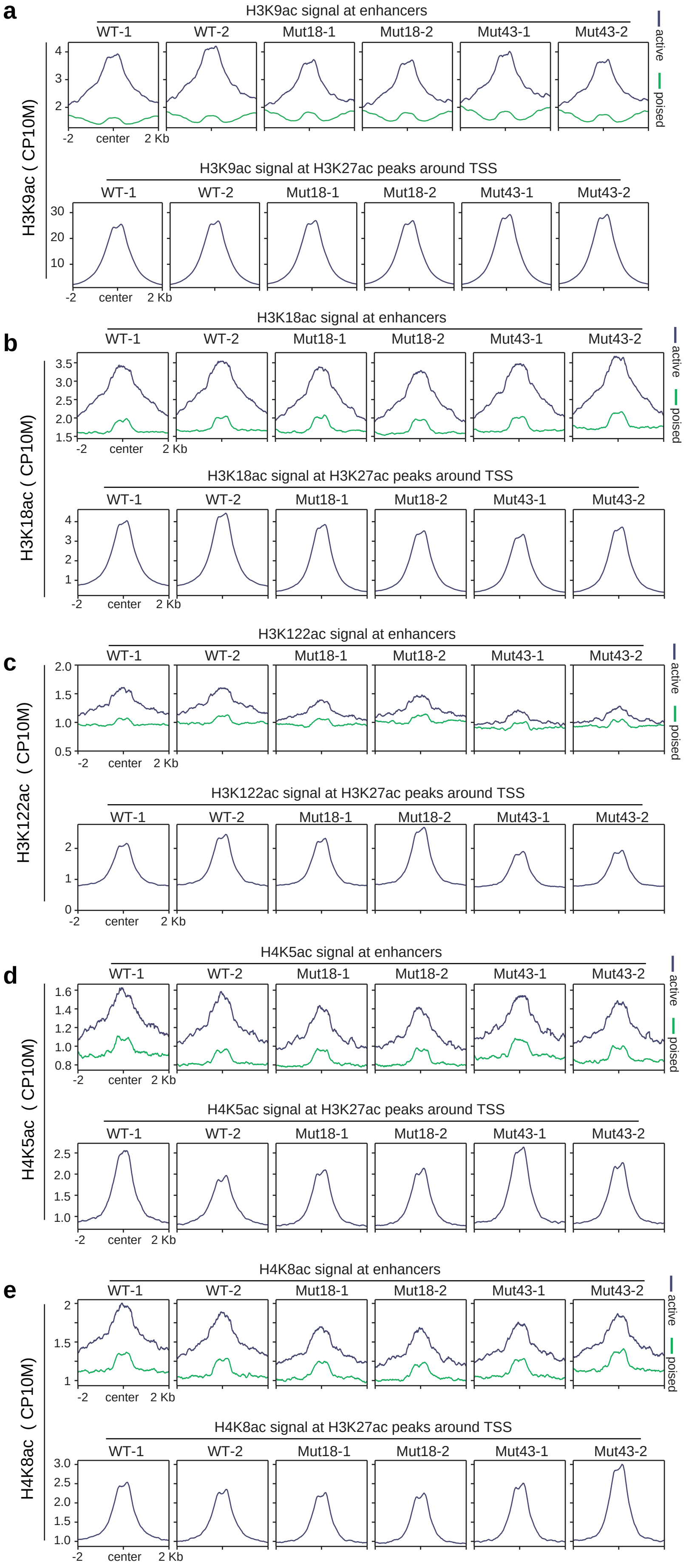

Figure S10. Averaged profiles of H3K9ac, H3K18ac, H3K122ac, H4K5ac, and H4K8ac at enhancers and H3K27ac-occupied promoters in WT and H3.3K27R mutant ESC lines. CP10M, counts per 10 million. (a) H3K9ac (**b**) H3K18ac. (**c**) H3K122ac. (**d**) H4K5ac. (**e**)H4K8ac.

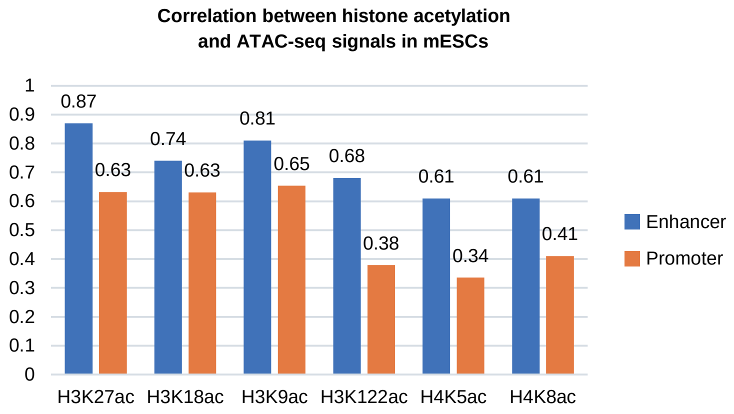

Figure S11. Barplots show Pearson correlation coefficients between ChIP-seq signal of histone acetylation and chromatin accessibility (ATAC-seq signal). Correlations were calculated for active enhancers (blue) and all promoter regions (upstream 1 kb and downstream 1 kb of TSS, orange) separately.
