# Supplementary material for: Histone H3K27 acetylation is dispensable for enhancer activity in mouse embryonic stem cells
Source: Genome Biol. 2020 Feb 21;21:45. doi: 10.1186/s13059-020-01957-w (PMC7035716; doi:10.1186/s13059-020-01957-w)
Supplement: Supplementary file 3 — Table S1. Sequencing depth and mapping efficiency of ChIP-seq and ATAC-seq experiments. Table S2. Peak numbers called in ATAC-seq and ChIP-seq experiments. Table S3. Stats for mRNA-seq experiments. Table S4 Sequences of DNA oligos used in this study. (PDF 459 kb) [file 13059_2020_1957_MOESM3_ESM.pdf]

Supplementary tables:

Table S1:

Cell line	Sample	Read pair#				
		raw	mapped	mapped%	unique	unique%
WT	input	23,885,044	22,297,522	93%	18,886,735	85%
	H3K4me3-1	18,400,948	15,856,970	86%	13,756,824	87%
	H3K4me3-2	24,210,337	20,841,804	86%	17,770,502	85%
	H3K9ac-1	36,856,117	33,788,837	92%	25,520,026	76%
	H3K9ac-2	35,782,354	32,690,051	91%	21,140,914	65%
	H3K18ac-1	26,602,778	24,452,925	92%	20,793,200	85%
	H3K18ac-2	35,051,721	32,259,163	92%	24,771,452	77%
	H3K27ac-1	26,213,330	23,261,109	89%	18,680,158	80%
	H3K27ac-2	23,817,781	20,971,586	88%	17,172,711	82%
	H3K27me3-1	21,182,877	18,072,640	85%	15,881,848	88%
	H3K27me3-2	30,896,481	26,807,861	87%	23,367,531	87%
	H3K122ac-1	25,526,181	24,064,148	94%	20,334,603	85%
	H3K122ac-2	28,132,501	26,424,974	94%	21,635,776	82%
	H4K5ac-1	10,202,048	8,208,236	80%	7,243,326	88%
	H4K5ac-2	27,756,953	25,851,115	93%	22,272,428	86%
	H4K8ac-1	26,441,271	24,223,164	92%	20,025,436	83%
	H4K8ac-2	24,531,619	22,963,683	94%	19,586,102	85%
	H3K4me1-1	39,145,770	36,192,206	92%	26,470,784	73%
	H3K4me1-2	43,760,021	40,531,836	93%	29,275,629	72%
	input-of-pol2	28,950,695	26,624,558	92%	20,841,209	78%
	pol2-1	37,324,312	31,746,072	85%	23,495,505	74%
	pol2-2	37,511,391	31,708,650	85%	23,241,124	73%
	ATAC-seq1	105,741,888	98,002,808	93%	79,751,127	81%
	ATAC-seq2	65,459,899	60,150,705	92%	50,509,381	84%
Mut18	input	25,047,450	23,402,416	93%	19,759,711	84%
	H3K4me3-1	25,154,054	21,544,279	86%	18,016,666	84%
	H3K4me3-2	23,555,478	20,167,634	86%	17,403,898	86%
	H3K9ac-1	38,319,700	34,981,303	91%	24,004,054	69%
	H3K9ac-2	32,085,509	29,261,277	91%	21,733,535	74%
	H3K18ac-1	24,436,257	21,844,233	89%	17,948,555	82%
	H3K18ac-2	28,967,864	25,949,570	90%	22,222,307	86%
	H3K27ac-1	27,050,019	22,944,017	85%	17,271,636	75%
	H3K27ac-2	26,379,413	21,453,774	81%	17,283,648	81%
	H3K27me3-1	31,116,112	26,616,229	86%	22,510,542	85%
	H3K27me3-2	21,720,186	18,309,446	84%	15,780,325	86%
	H3K122ac-1	23,911,027	22,447,690	94%	18,104,337	81%
	H3K122ac-2	19,404,681	18,133,174	93%	14,869,906	82%

	H4K5ac-1	26,559,917	24,669,036	93%	20,614,095	84%
	H4K5ac-2	24,233,913	22,608,068	93%	19,244,445	85%
	H4K8ac-1	25,931,779	24,377,372	94%	20,226,345	83%
	H4K8ac-2	24,386,907	22,922,680	94%	19,418,963	85%
	H3K4me1-1	38,261,768	35,861,323	94%	26,216,146	73%
	H3K4me1-2	35,391,427	33,209,335	94%	24,935,277	75%
	input-of-pol2	31,754,163	28,895,601	91%	22,398,658	78%
	pol2-1	42,213,190	37,991,833	90%	27,477,254	72%
	pol2-2	45,476,715	40,938,546	90%	30,469,917	74%
	ATAC-seq1	61,864,250	57,018,686	92%	47,889,152	84%
	ATAC-seq2	73,485,014	67,547,027	92%	55,990,092	83%
Mut43	input	29,165,368	27,301,284	94%	22,201,207	81%
	H3K4me3-1	20,008,629	16,806,529	84%	14,955,107	89%
	H3K4me3-2	28,615,627	24,064,741	84%	21,093,431	88%
	H3K9ac-1	30,867,020	27,779,347	90%	20,343,129	73%
	H3K9ac-2	37,152,752	33,565,616	90%	23,976,645	71%
	H3K18ac-1	25,940,870	23,003,419	89%	20,185,611	88%
	H3K18ac-2	19,644,466	17,386,981	89%	14,867,662	86%
	H3K27ac-1	24,307,376	19,171,901	79%	15,585,540	81%
	H3K27ac-2	25,423,415	20,192,052	79%	15,502,793	77%
	H3K27me3-1	26,340,713	21,490,302	82%	19,197,118	89%
	H3K27me3-2	33,141,588	27,076,506	82%	23,897,351	88%
	H3K122ac-1	26,407,881	24,739,382	94%	20,555,031	83%
	H3K122ac-2	25,899,274	24,308,537	94%	20,081,992	83%
	H4K5ac-1	19,529,320	17,846,303	91%	15,582,343	87%
	H4K5ac-2	29,939,557	27,271,938	91%	23,190,162	85%
	H4K8ac-1	23,366,480	21,778,950	93%	18,968,748	87%
	H4K8ac-2	23,271,154	21,634,973	93%	18,181,882	84%
	H3K4me1-1	35,732,218	33,351,569	93%	26,156,161	78%
	H3K4me1-2	36,196,348	33,811,850	93%	26,041,749	77%
	input-of-pol2	29,557,714	27,019,772	91%	20,555,158	76%
	pol2-1	50,923,871	45,057,465	88%	33,470,838	74%
	pol2-2	34,469,236	30,661,514	89%	21,258,511	69%
	ATAC-seq1	70,521,889	65,068,365	92%	54,762,867	84%
	ATAC-seq2	58,527,893	54,955,717	94%	44,563,894	81%

Table S1. Sequencing depth and mapping efficiency of ChIP-seq and ATAC-seq experiments.

Table S2:

ChIP samples	replicates	WT	Common (WT)	Mut18	Common (Mut18)	Mut43	Common (Mut43)
H3K27ac	rep1	43,873	32,442	11,175	9,788	18,001	13,142
(q<1e-2)	rep2	43,625		16,737		17,547	
H3K4me1	rep1	67,593	53,858	57,794	42,873	70,499	58,926
(q<1e-3)	rep2	67,024		64,026		76,652	
H3K4me3	rep1	32,098	30,893	37,865	34,848	36,563	33,648
(q<1e-6)	rep2	33,891		38,449		40,751	
H3K27me3	rep1	17,623	8,436	12,071	8,635	22,419	14,374
(q<1e-2)	rep2	12,180		16,986		20,520	
ATAC-seq	rep1	78,539	62,507	63,591	58,746	68,634	59,030
(q<1e-6)	rep2	67,419		59,814		65,802	

Table S2. Peak numbers called in ATAC-seq and ChIP-seq experiments. Common peaks between replicates are also shown for comparison.

Table S3:

RNA sample	Read Pair#	Mouse (mm10)		*Drosophila* (dm6)	
		Uniquely mapped	gene# (read count>=10)	Uniquely mapped	gene# (read count>=10)
WT-1	20,752,313	16,980,310	13,893	1,589,279	6,413
WT-2	20,711,959	16,911,999	13,745	1,633,919	6,412
Mut18-1	20,513,081	16,846,545	13,643	1,600,657	6,391
Mut18-2	20,532,932	16,830,430	13,583	1,606,794	6,387
Mut43-1	20,576,702	16,823,350	13,560	1,620,143	6,411
Mut43-2	20,556,551	16,912,099	13,546	1,572,220	6,408

Table S3. Stats for mRNA-seq experiments. Sequencing depth and gene coverage are shown for both mouse ESC samples and *Drosophila* S2 cell spike-ins.

Table S4:

	**sgRNA targeting sequence with PAM (5'-3')**
H3f3a sgRNA1	GCTTAATTAGCGCTCGACAC **CGG**
H3f3a sgRNA2	TACTAGTTGACTATACTAGA **GGG**
H3f3b sgRNA1	TGGTGGCCAGCTGTTTGCGG **GGG**
H3f3b sgRNA2	AAGCGCGCCCTCTACCGGCG **GGG**
	**Homologous arm amplification primers (5'-3')**
H3f3a HDR left-F	GGCTCCTCTTTCTTCGGTGAAATCC
H3f3a HDR left-R	GGCTGGAGAGATTGCTCAGCAG
H3f3a HDR right-F	CTCTAGTATAGTCAACTAGTAATAGGACACG
H3f3a HDR right-R	GGGTCTTATCTCCCAGCATTCTAGC
H3f3b HDR left-F	CGAAGTACGGCCTTTGTGTCG
H3f3b HDR left-R	GTCAGGGAGAAGGGAGAGAGC
H3f3b HDR right-F	CGTTGACGCCTTCCTTCTTCTG
H3f3b HDR right-R	CACATAGTGTATTCTATAGCTGCCAGG
	**K27R point mutation and PAM mutation primers (5'-3')**
H3f3a K27R AAG-AGA mut-F	TCGCAGAAGTGCGCCCTC
H3f3a K27R AAG-AGA mut-R	CACTTCTGCGAGCGGCTTTTG
H3f3a gRNA1 PAM mut-F	GGCGCCAGTCGAGCGCTAATTAAGCCTG
H3f3a gRNA1 PAM mut-R	ACTGGCGCCGCCCGGCCCACCCAGCA
H3f3b K27R AAA-AGG-F	CGGCTCGGAGGAGCGCGCCCTCTAC
H3f3b K27R AAA-AGG-R	GCGCGCTCCTCCGAGCCGCCTTGG
H3f3b gRNA1 PAM mut-F	CCCGAGGAAACAGCTGGC
H3f3b gRNA1 PAM mut-R	TGTTTCCTCGGGGCTTTCCCAC
H3f3b gRNA2 PAM mut-F	CCGGAGGCGTGAAGAAGCCTCACCGCTAC
H3f3b gRNA2 PAM mut-R	CACGCCTCCGGTAGAGGGCGCGCT
	**Genotyping primers (5'-3')**
H3f3a K27R test-F	GGGGTAAGTTTCTCCGTTTGC
H3f3a K27R test-R	CCCTTAATATAACTTCGTATAATGTATGC
H3f3b K27R test-F	ACCAAGGCGGCTCGGAGG
H3f3b K27R test-R	GCCTCCTGTTGAGGACAAGAGC
	**Genomic DNA amplification primers for Sanger sequencing (5'-3')**
H3f3a K27R seq amp-F	CGCGCCCTTCCAAACTAATTATATAAC
H3f3a K27R seq amp-R	GCTGGTTGTGGTATTCACACTTG
H3f3a K27R seq	CGTGTCCTATTACTAGTTGACTATACTAG
H3f3b K27R seq amp-F	CCAATCAGAAGAGCTCCCTAGTGC
H3f3b K27R seq amp-R	GCCTCCTGTTGAGGACAAGAGC
H3f3b K27R seq	GGCGCAGCCTGAGTCATTAG
	**Reverse transcription primers for Sanger sequencing (5'-3')**
H3f3a cDNA-F	CCTCGGTGTCAGCCATCTTTC

H3f3a cDNA-R	CCCTCATAGTGGACTCTTAAGCAC
H3f3b cDNA-F	CCGATTGCGGCTCTTGTTCG
H3f3b cDNA-R	ACCGCTTCAACTTAAGCTCTCTC

Table S4. Sequences of DNA oligos used in this study.
