# Supplementary material for: Histone H3K27 acetylation is dispensable for enhancer activity in mouse embryonic stem cells
Source: Genome Biol. 2020 Feb 21;21:45. doi: 10.1186/s13059-020-01957-w (PMC7035716; doi:10.1186/s13059-020-01957-w)
Supplement: Supplementary file 4 — Review history. [file 13059_2020_1957_MOESM4_ESM.docx]

Review History

**First round of review**

**Reviewer 1**

**Are you able to assess all statistics in the manuscript, including the appropriateness of statistical tests used?**

Yes, and I have assessed the statistics in my report.

**Comments to author:**

Enhancer function is critical for spatiotemporal gene expression. This inspires researchers to probe the features of enhancers and also to map or predict enhancer genome-wide. Ever since the first reports declaring H3K27ac as the marker of active enhancers (Creyghton M.P etal.,2010), H3K27ac had been frequently used as a chromatin feature to predict active enhancers both in animals and plants. Besides, H3K27ac is often found to be tightly linked to active gene expression. Recently, H3K27ac was found to locate outside of active enhancer regions in Arabidopsis (Wenhao Yan etal., 2019). The functional basis of H3K27ac in active enhancer activity remains mystery. The manuscript from Tiantian ZHANG etal deliveries the messages that H3K27ac may be neither necessary for enhancer function or for active gene expression. They created a mutant that loses large proportion of H3K27ac both in active enhancers and transcription starting sites but shows little enhancer activity and gene expression change. By investigating the change of other modifications, the authors conclude that there might be compensation effects among multiple acetylations. The findings here are particularly of interest to the community and would bring big impact to the field, for instance changing the strategy of genome-wide enhancer mapping. I would be happy to recommend the work to be published in Genome biology if my following concerns could be addressed:
1.     Regarding H3K27ac level in both H3.3K27R mutants, compared to wt, the decrease in both mutant cases is very mild (around 20% by figure 1a?), doesn't seem to meet the diminished level by ChIP-Seq from Figure1 d and e. Also, the report from Mary Leatham-Jensen et al.,2019 showed around 5-fold decrease in H3K27ac levels between the H3.3BK27R mutant and wild type. Could the author quantify the decrease in Figure 1a? Is there any explanation for such mild difference between the mutant and the wt? Such mild difference between mutant and wild type may harm the conclusion of the manuscript.
2.     Is there any reason why way bigger proportion of enhancer-overlapping H3K27ac showed reduced H3K27ac level as compared to promoters in the mutant (80% vs 30%)?
3.     The authors showed that K18ac, K23ac, K122ac and K4 acetylation are all comparable between wild type and the mutants. What about H3K9ac, which is another frequently used mark for active enhancers?

**Reviewer 2**

**Are you able to assess all statistics in the manuscript, including the appropriateness of statistical tests used?**

No, I do not feel adequately qualified to assess the statistics.

**Comments to author:**

The manuscript by Zhang et al studies the role of H3K27 acetylation in enhancer maintenance. By substituting lysine 27 in histone variant H3.3 with arginine in mouse embryonic stem cells, they find that diminishing the vast majority of H3K27ac at enhancers has little effect on other enhancer features, including chromatin accessibility, H3K4me1 levels, as well as acetylation at some other histone lysine residues. Consequently, global gene expression is largely undisturbed in these mutant cells. Overall, this is a well-designed and executed study, with clear results supporting the conclusions. There are few points need to be clarified.  
1.     Previous studies by Banaszynski and others have shown that H3.3 is not essential for mouse ESC self-renewal and differentiation. Although H3K27ac levels decrease upon H3.3 depletion, other active marks, such as H3K4me3 and H3K26me3, are largely unaffected. Therefore, the finding from the current study in that other enhancer features are not affected by H3.3K27 substitution in mESC is somewhat not unexpected. I realize that it is probably beyond the scope of the current study, however an interesting question is that whether such an H3.3K27R mutation could have a stronger effect in somatic cells or some cancer cell lines where H3.3 is important. This point need to be at least discussed in this manuscript.
2.     In the title, it should be spelled out as "mouse ESC", as we do not know whether the same mutation in ESCs from other species will show the same phenotypes.  
3.     Although H3.3K27R mutation does not affect enhancer maintenance during mESC self-renewal, does it affect establishment of new enhancers during cell differentiation?
Minor points:
1. Associating active enhancers to genes by one-dimension distance has low accuracy. Combining histone acetylation signals and chromatin accessibility data may give a better prediction power for enhancer-promoter pairs (DOI: 10.1038/s41588-019-0538-0).
2. Alternatively, since multiple acetylation ChIP-seq data have been generated, the authors could determine the contribution of each histone acetylation to enhancer or promoter activity using linear model.
3. In Figure 2h-i, the mean profile of Pol II in the mutant cells shows slightly higher density towards gene bodies, are the changes in Pol II pausing index corresponding with changes of H3K27ac?  Are global levels of elongating Pol II and total Pol II proteins increased in mutation cells?

**Authors Response**

**Point-by-point responses to the reviewers’ comments:**

Comment

*Response*

Reviewer #1: Enhancer function is critical for spatiotemporal gene expression. This inspires researchers to probe the features of enhancers and also to map or predict enhancer genome-wide. Ever since the first reports declaring H3K27ac as the marker of active enhancers (Creyghton M.P et al.,2010), H3K27ac had been frequently used as a chromatin feature to predict active enhancers both in animals and plants. Besides, H3K27ac is often found to be tightly linked to active gene expression. Recently, H3K27ac was found to locate outside of active enhancer regions in Arabidopsis (Wenhao Yan et al., 2019). The functional basis of H3K27ac in active enhancer activity remains mystery. The manuscript from Tiantian ZHANG et al deliveries the messages that H3K27ac may be neither necessary for enhancer function or for active gene expression. They created a mutant that loses large proportion of H3K27ac both in active enhancers and transcription starting sites but shows little enhancer activity and gene expression change. By investigating the change of other modifications, the authors conclude that there might be compensation effects among multiple acetylations. The findings here are particularly of interest to the community and would bring big impact to the field, for instance changing the strategy of genome-wide enhancer mapping.

*We thank the reviewer for pointing out the significance of our study and for providing insightful suggestions.*

I would be happy to recommend the work to be published in Genome biology if my following concerns could be addressed:

1. Regarding H3K27ac level in both H3.3K27R mutants, compared to wt, the decrease in both mutant cases is very mild (around 20% by figure 1a?), doesn't seem to meet the diminished level by ChIP-Seq from Figure1 d and e. Also, the report from Mary Leatham-Jensen et al.,2019 showed around 5-fold decrease in H3K27ac levels between the H3.3BK27R mutant and wild type. Could the author quantify the decrease in Figure 1a?

*We quantified the relative levels of H3K27ac in H3.3K27R mutant cell lines compared to wildtype mouse ESCs by Image J for two independent western blots. Our results suggest an approximately 50% reduction of H3K27ac levels in H3.3K27R mutant cell lines compared to wildtype, which is within a comparable range with a recent publication reporting an approximately 40% reduction on H3K27ac levels in H3.3- depleted mouse ESCs (Martire et al., Nature Genetics, 2019). This differs from a nearly 5-fold decrease in H3K27ac levels in H3.3BK27R male flies reported by Leatham-Jensen et al., likely due to species-specific regulations. Considering that the semiquantitative nature of Western blots, we did not include the quantification into the manuscript.*

Is there any explanation for such mild difference between the mutant and the wt? Such mild difference between mutant and wild type may harm the conclusion of the manuscript.

*We thank the reviewer for raising this point, which is likely shared by many readers.*

*We only mutated H3.3. The majority of H3 histones in rapid dividing cells, such as ES cells that we used, are canonical histones (H3.1 and H3.2). These histones can still be acetylated at K27 in our mutant cells, therefore the bulk change around 50% is pretty high, and is consistent with the fact that H3.3 histones are enriched for histone modifications associated with active transcription. To express this clearer, we rephrased our text as the following: We first measured the global H3K27ac level by Western blot analysis and observed a moderately reduced bulk H3K27ac level in mutant cells (Fig. 1a), which is expected because mouse ESCs contain much higher levels of canonical H3 histones, which remain possible to be acetylated at K27.*

*Moreover, the impact of H3.3K27R mutation at enhancer K27ac level is much stronger likely because enhancers are highly enriched for H3.3 and K27ac. Our ChIP-seq results in Fig. 1 and Fig. S6 also revealed that H3K27ac was nearly wiped out at active enhancers, suggesting that the vast majority of enhancer H3 histones are H3.3.*

2. Is there any reason why way bigger proportion of enhancer-overlapping H3K27ac showed reduced H3K27ac level as compared to promoters in the mutant (80% vs 30%)?

*There are two possible reasons. First, the turnover rate of H3.3 is faster at active enhancers than at the TSS regions (Deaton et al., eLife, 2016; Kraushaar et al., Genome Biology, 2013). Moreover, H3.3 occupancy around active enhancers is much higher than that at TSS regions. We analyzed recently published H3.3 ChIP-seq data (Wang et al., BMC Biology, 2018) and compared the H3.3 signals around active enhancers with that around H3K27ac-marked TSSs. The result presented in the new Fig. S4, together with H3K27ac ChIP-seq results in Fig. 1, suggest that the vast majority of H3 histones at active enhancers are H3.3, instead of H3.1 or H3.2; whereas H3.1 and H3.2 contribute to a significant portion of H3 histones at promoters.*

3. The authors showed that K18ac, K23ac, K122ac and K4 acetylation are all comparable between wild type and the mutants. What about H3K9ac, which is another frequently used mark for active enhancers?

*We performed ChIP-seq to profile the genome-wide distribution of H3K9ac in wild-type and H3.3K27R mutant cell lines. As shown in the new Fig. S10a, the effect on H3K9ac distribution was minimal. These results further support our conclusion that the maintenance of enhancer activity does not solely depend on H3K27ac; instead, it depends on the combined effects of histone acetylation at different lysine residues.*

Reviewer #2:

The manuscript by Zhang et al studies the role of H3K27 acetylation in enhancer maintenance. By substituting lysine 27 in histone variant H3.3 with arginine in mouse embryonic stem cells, they find that diminishing the vast majority of H3K27ac at enhancers has little effect on other enhancer features, including chromatin accessibility, H3K4me1 levels, as well as acetylation at some other histone lysine residues. Consequently, global gene expression is largely undisturbed in these mutant cells. Overall, this is a well-designed and executed study, with clear results supporting the conclusions.

*We appreciate the reviewer’s affirmative comments and insightful suggestions, which are very helpful in improving our manuscript and projecting future study.*

There are few points need to be clarified.

1. Previous studies by Banaszynski and others have shown that H3.3 is not essential for mouse ESC self-renewal and differentiation. Although H3K27ac levels decrease upon H3.3 depletion, other active marks, such as H3K4me3 and H3K26me3, are largely unaffected. Therefore, the finding from the current study in that other enhancer features are not affected by H3.3K27 substitution in mESC is somewhat not unexpected. I realize that it is probably beyond the scope of the current study, however an interesting question is that whether such an H3.3K27R mutation could have a stronger effect in somatic cells or some cancer cell lines where H3.3 is important. This point need to be at least discussed in this manuscript.

*In the Banaszynski paper, both alleles of* h3f3b *were knocked out, in addition,* h3f3a *was knocked down with shRNA. On the other hand, the Magnuson lab reported that the full knockout of both* H3.3a *and* H3.3b *genes are cellular lethal in mouse ES cells.*

*We cited their study in the Background part and referred as “complete loss of H3.3 in mice is embryonic lethal at an early stage and even cellular lethal as a result of mitotic defects”. Thus, H3.3 genes appear to be rather important even in ES cells. In addition, as suggested, we added the following sentence into the discussion: Our results clearly indicate that the depletion of H3K27ac does not affect enhancer activity in mouse ESCs, whether it may have a stronger impact in other cells types such as somatic cells and cancer cells can be addressed using the same approach in the future.*

2. In the title, it should be spelled out as "mouse ESC", as we do not know whether the same mutation in ESCs from other species will show the same phenotypes.

*We agree this is more accurate and we have rephrased the title accordingly.*

3. Although H3.3K27R mutation does not affect enhancer maintenance during mESC self-renewal, does it affect establishment of new enhancers during cell differentiation?

*We appreciate the reviewer for pointing out this point, and we intend to address this in a future study for reasons that will be explained below. Indeed, we have analyzed the embryoid body (EB) differentiation process with the H3.3K27R mutant cells, and performed RNA-seq experiments. We did observe some defects of H3.3K27R mutant cells during EB differentiation at both morphological and gene expression levels.*

*However, we think the differentiation defect is likely associated with some unknown event that we are currently investigating on, rather than a role of H3.3K27ac in regulating enhancer establishment during cell differentiation. Although this is our current opinion, we cannot completely rule out the abovementioned possibility, therefore we would like to carefully restrict our conclusion to the maintenance of enhancer activity, and we prefer to leave the establishment issue in a future study.*

Minor points:

1. Associating active enhancers to genes by one-dimension distance has low accuracy. Combining histone acetylation signals and chromatin accessibility data may give a better prediction power for enhancer-promoter pairs (DOI: 10.1038/s41588-019-0538-0).

*We agree that this is a better approach. In the revised manuscript, we used the activity-by-contact (ABC) model developed by the referred report (Fulco et al., Nature Genetics, 2019) to predict the enhancer-gene connections in the wild-type mouse ESCs, and re-analyzed our RNA-seq data. Applying ABC model enabled us to link much more genes (N = 8,911) to H3K27ac-marked active enhancers compared with our previous analysis (N = 2,395). With the new method, we observed 53 upregulated and 195 downregulated genes in H3.3K27R mutant ESCs (new Fig. 2b). Similarly, H3K27ac at enhancers of both upregulated and downregulated genes was decreased as we have shown in our previous analysis (new Fig. 2c).*

2. Alternatively, since multiple acetylation ChIP-seq data have been generated, the authors could determine the contribution of each histone acetylation to enhancer or promoter activity using linear model.

*Due to the difference in antibody affinities and ChIP efficiency, it is difficult to directly compare ChIP-seq results generated with antibodies against acetylation at different lysine residues, especially if we want to precisely quantify their contributions. On the other hand, it is highly feasible to compare data generated with the same antibody across the genome. Therefore, we took an alternative approach and used chromatin accessibility as a proxy for the activity of enhancers and promoters and determined the Pearson correlation of each histone acetylation with chromatin accessibility (ATAC-seq) at enhancers and promoters (Fig. S11). This analysis revealed that indeed H3K27ac is the best marker for the accessibility of enhancers, once again supporting a role of H3K27ac as a well-defined indicator of active enhancers. However, our analysis also indicates that H3K27ac is not the sole marker that display a positive correlation with enhancer accessibility. Acetylation at other lysine residues can also nicely display a positive correction with enhancer accessibility. This is in agreement with our main conclusion that H3K27ac is and remains to be the best marker for enhancer indication, but functionally it has to work in concert with acetylation events on other histone lysine residues.*

3. In Figure 2h-i, the mean profile of Pol II in the mutant cells shows slightly higher density towards gene bodies, are the changes in Pol II pausing index corresponding with changes of H3K27ac? Are global levels of elongating Pol II and total Pol II proteins increased in mutation cells?

*We thank the reviewer for carefully analyzing our data. RNA Pol II signal is comparable at TSS regions in both WT and mutant cells, reflecting undisturbed transcription initiation. We think the elevated Pol II signals towards gene bodies are unlikely caused by the loss of H3K27ac in the mutant cells, because H3K27ac is not enriched in the gene bodies and the 3’-ends in wild-type ES cells. We are highly intrigued by this phenomenon. We hope the reviewers will agree with us that this is out of the scope of our current study, and we will devote ourselves into this interesting future direction.*

**Second round of review**

**Reviewer 1**

I appreciate the authors for their efforts to provide new data and the nice discussions to address my concerns. I am happy with the response and I would recommend acceptance of the manuscript.

**Reviewer 2**

The authors have adequately addressed all my concerns. I thus recommend publication at Genome Biology.
